# "The case for clean indoor air"

**DOI:** 10.1186/1477-3163-5-8

**Published:** 2006-02-10

**Authors:** Fred M Jacobs

**Affiliations:** 1 Commissioner, New Jersey Department of Health and Senior Services, State of New Jersey, Trenton, NJ 08625 USA

In 2004, a Tsunami in South Asia killed more than 180,000 people and the world recoiled in shock and disbelief. In 2005 an earthquake in Pakistan killed close to 100,000 and the world was devastated. These are truly terrible disasters that continue to claim lives, and we are rightly horrified by their scope and scale.

Yet, every year 400,000 Americans die from smoking cigarettes and few people notice, and fewer still are standing up to try to save those lives. We must begin to see the tragedy of smoking-related deaths as a challenge as great as any natural disaster.

Tobacco is a legal product which, when used as directed, leads to the death or disability of the user. I cannot think of any other product like that on the market today.

More people die from tobacco than alcohol, AIDS, car crashes, illegal drugs, murders and suicides combined. It is the leading preventable cause of death in the United States.

In my view, smoking and exposure to second-hand smoke are the most serious public health threats that we face today. I say this not only as Commissioner of New Jersey's Department of Health and Senior Services, but also as a doctor who specialized in pulmonary disease for 40 years.

In practice I saw thousands of patients, many of whom were non-smokers, suffering from illnesses caused by tobacco. This damage was not just in the lungs, but takes root in nearly every organ in the body.

Smoking causes about 90 percent of lung cancer deaths in men and almost 80 percent in women, according to the Surgeon General's 2004 report. Other cancers caused by smoking include cancers of the larynx, bladder, stomach, mouth, cervix, kidney and pancreas, and acute myeloid leukemia. Smoking is also a powerful contributor to bronchitis and emphysema, stroke, cardiovascular disease, pneumonia, pregnancy complications, premature and still births and Sudden Infant Death Syndrome.

Tobacco creates a pervasive health problem for smokers, but it doesn't stop there. Secondhand smoke is the third leading cause of preventable death in the United States, killing 53,000 non-smokers each year. These people choose not to smoke, yet die from the consequences of the smoking behavior of others.

Among children less than 18 years old, an estimated 22 percent are exposed to secondhand smoke in their homes. This exposure results in a variety of upper and lower respiratory tract illnesses. Second-hand smoke in children can exacerbate asthma and cystic fibrosis. We also know from the Environmental Protection Agency that it is associated with an estimated 8,000 to 26,000 new asthma cases in children each year.

And second-hand smoke has a very significant – and negative – effect on infants. Smoking by the mother is a cause of Sudden Infant Death Syndrome. Compared with unexposed infants, babies exposed to secondhand smoke after birth are at twice the risk for SIDS, and infants whose mothers smoked before and after birth are at three to four times greater risk.

For pregnant women the nicotine in cigarettes causes constrictions in the blood vessels of the umbilical cord and uterus, thereby decreasing the amount of oxygen available to the fetus. Nicotine can also reduce the amount of blood in the fetal cardiovascular system. It is also found in breast milk. Babies of women who smoked during pregnancy have lower birth weights. Women who smoke during pregnancy reduce their babies' lung function.

Pregnant smokers are also at a higher risk for premature rupture of membranes before labor. This makes it more likely that a smoker will carry her baby for a shorter than normal gestation period.

Knowing this, how can we do nothing? How can we stand by with the knowledge that from the womb to the grave we are shortening and reducing the quality of our lives? Why are we moved by the deaths of thousands in natural disasters and not by the deaths we could easily prevent by reducing tobacco use in the workplace?

Secondhand smoke is itself a class A carcinogen. It causes more cancer deaths than asbestos, arsenic, radiation, pesticides, benzene, vinyl chloride, hazardous waste sites, contaminated sludge, mining waste and chemicals found in drinking water combined.

There are rules regarding exposure in the workplace for cancer causing chemicals, yet not for the cancer causing chemicals in tobacco smoke. We don't abide asbestos flying around in our restaurants or bars or schools, yet we allow second-hand smoke to pollute our air.

Patrons in bars and restaurants are exposed to these toxic fumes for perhaps an hour or two a few times a month, but the employees suffer this exposure for an entire working day, five or more days a week.

I am convinced that most people are not aware of how grave a health risk they take each time they enter a restaurant or bar that allows smoking, or the benefits they'll find if smoking were banned.

Prior to the smoking ban in 2003, New York bar and restaurant employees reported being exposed to 12 hours of smoke over a four-day period; that figure dropped to 12 minutes in 2004.

Bar and restaurant workers in New York are suffering fewer sore throats and runny noses since that state's workplace smoking ban went into effect, according to findings published in the August 2005 issue of Tobacco Control.

Since Rhode Island's smoke-free law took effect in March 2005, the average number of tiny particles suspended in the air fell 96 percent, according to Rhode Island's state-sponsored study. The researchers focused on particles smaller than 2.5 microns, a size that includes fumes from second-hand tobacco smoke.

Many arguments against a public smoking ban are based on the notion that going smoke free will have a tremendous, negative impact on business revenues, forcing them to either raise prices to compete, or to close their doors.

That issue was raised in New York City when its Smoke-Free Air Act went into effect more than two years ago.

The fears proved unfounded. There was no negative impact on jobs or revenues. In fact, the economic situation for bars and restaurants improved, according to a report by the New York City Economic Development Corporation and the departments of Taxation, Small Business Services and Health and Hygiene.

Business tax receipts in restaurants and bars were up 8.7 percent a year after enactment. Employment in restaurants and bars increased by 10,600. More establishments have liquor licenses than before the law went into effect. At the same time air quality in bars and restaurants improved dramatically, and one hundred and fifty thousand fewer people are exposed to smoke on the job.

California passed its first law restricting smoking in workplaces in 1994. On January 1, 1998, California became the first state in the nation to eliminate smoking in virtually all indoor workplaces, including bars, taverns and gaming clubs.

The California Department of Health Services concluded in 2001 that, "As sales tax data accumulated from 1998 forward, following...implementation...in bars, taverns and gaming clubs, economic fear proved groundless."

In Delaware, business remained steady more than a year after the state's Clean Indoor Air Act went into effect. Data shows that the number of restaurant, tavern and tap room licenses increased in the year since the law took effect. Employment in food service and drinking establishments also increased.

It is past time to provide for smoke free indoor air. The public's health demands that we take action. It is an idea whose time has come!

As New Jersey's Commissioner of Health and Senior Services, it is my obligation, and indeed my duty to support the elimination of smoking in indoor public places as a matter of great importance to the protection of the public health. In fact, it would be unconscionable for me not to.

I am pleased that the New Jersey Senate and Assembly passed the Clean Indoor Air Act just this week. Governor Richard J. Codey has pledged to sign the bill, and improve the health of tens of thousands of New Jerseyans.

We are the tenth state to make such a ban, and I am confident that we will not be the last. Protecting the health of employees, children and non-smokers alike from the ill-effects of tobacco is vitally important to the public health here in New Jersey, the United States and the world.

